# Inductively coupled, mm-sized, single channel optical neuro-stimulator with intensity enhancer

**DOI:** 10.1038/s41378-019-0061-6

**Published:** 2019-06-03

**Authors:** Wasif Khan, Yaoyao Jia, Fatma Madi, Arthur Weber, Maysam Ghovanloo, Wen Li

**Affiliations:** 1 Department of Electrical and Computer Engineering, Michigan State University, East Lansing, MI USA; 2 School of Electrical and Computer Engineering, Georgia Institute of Technology, Atlanta, GA USA; 3 Department of Physiology, Michigan State University, East Lansing, MI USA

**Keywords:** Electrical and electronic engineering, Micro-optics

## Abstract

We introduce a single channel neuro-stimulator consisting of a reflector-coupled microscale light emitting diode (µLED) with an integrated mm-sized wireless receiver (Rx) coil for free-floating, battery-free, untethered optogenetics neuromodulation. The system utilizes a two-coil inductive link to deliver instantaneous power at a low operating frequency (<100 MHz) for continuous optical stimulation with minimized invasiveness and tissue exposure to electromagnetic radiation. Coupling a microscale reflector to the µLED provides significant light intensity enhancement compared to a bare µLED. Our activated stimulators have an operational temperature increase of <1 °C, well below the safety limit of biomedical implants. In vivo experiment and histological analysis verify the efficacy of wireless optical stimulation in the primary visual cortex of rats, using c-Fos biomarker as a reporter of light-evoked neuronal activity.

## Biotechnology: Wireless power lights up tiny neural stimulators

Miniature coils smaller than a grain of rice can improve the comfort of implantable optical devices that target neurons. Optogenetic technology uses pulses of light to turn protein expression in genetically modified neurons on or off. Controlling and collecting data from these devices usually requires bulky cables and batteries, but Wasif Khan from Michigan State University in the United States and colleagues have now developed a completely wireless prototype. The team coupled a microscale light-emitting diode with two millimeter-scale coils, creating an inductive charging system that can deliver instantaneous power at biologically safe frequencies. Experiments with live rat models showed that this wireless setup could stimulate neurons located in the visual cortex while keeping temperature increases well below 1 °C, a critical safety threshold for biomedical implants.

## Introduction

Rapid progress in neuroscience discoveries has been achieved by successful incorporation of semiconductor devices with/in biological systems and by their successful transformation into clinical devices^1^. In contrary to current prevalent pharmacological approaches, medicine and therapy for future have a strong potential to be revolutionized by implantable “electroceuticals” which essentially target the neural pathways, in both the central and peripheral nervous systems for therapeutic intervention^2^. Optogenetics, the technology of delivering light to tissues of interest while collecting readouts from the cells using targeted control tools^3^ has become a prominent method for recent neuroscience discoveries and an effective method towards future implantable therapeutic approaches. With the ability to implant/insert optical sources, recording electrodes, sensors, and other components into intended locations of the brain, interesting and promising diagnostics and therapeutic capabilities could be generated^2,4^. Cells that specifically drive or inhibit fundamental functions such as hunger, thirst and energy balance^5–8^, respiration^9^ have been identified with their activity patterns. With the help of optogenetics, it was also possible to study the transmission of primary sensory information to the brains regarding olfactory^10^, visual^11^, and auditory^12^ domains.

A major challenge for using optogenetics in behavioral studies is to effectively and practically deliver sufficient light to the target neural population without altering the natural behavior of experimental animals. Early development of optical stimulation tools required penetrating optical fibers with interconnections to externally located electronic control and light sources (i.e., lasers)^3^. Recent demonstration of surface mounted optical sources and electronics (i.e., LED and Bluetooth data transmission systems) also exposes the necessity of being connected with batteries or outside power sources^13^. Several optical implants comprising of bulky batteries and wired interfaces have been reported, which unfortunately increase the size and weight of the system and thereby induce difficulties with in vivo experimentations as well as the long-term stability and reliability of the implant^13–16^. These tethered systems also require manual and physical attachment/detachment of an optical fiber or batteries before behavioral testing, thereby limiting the environments in which freely behaving optogenetic experiments and data collection could be performed.

To address this issue, some groups have implemented fiber rotation techniques during animal movements^17^. Although this approach has improved the ease of device attachment and detachment, the tethering still increases chronic physical trauma at the implanted site that could interfere with or alter the outcomes of behavioral studies. Furthermore, surface-mounted stimulation sources and data acquisition systems suffer from the deficiency in penetrating into the volumetric depths of the tissues, which poses a significant challenge in their operations^18^.

To eliminate the deficiencies of wire tethering, several wireless powered optical stimulation sources have been demonstrated in previous reports and rapid progress has been observed on the evolvement of wireless powered stimulators. Pioneering work by researchers on RF mid-field and far-field power transfer has led to several optimized designs of neuro-stimulators^19–22^. Wireless stimulators operating at high frequencies (910 MHz or 1.5 GHz)^1,19^ were implemented to achieve the desired untethered power transfer. However, the high operating frequency range of these systems is unsafe for nerve vitality and increases the risk of over-exposure of electromagnetic radiation and microwave-induced heating of nerve tissues^23^. Another alternative approach is ultrasonic power transfer for implants^24,25^ with energy focusing at large depths^26–29^, superior transduction efficiency^30,31^ and low tissue attenuation (∼0.5–1 dB/cm/MHz)^32^. However, a fully implantable miniaturized high voltage (HV) stimulator providing precise control of stimulation parameters has not yet been reported^33^.

To address the above challenges, an ideal wireless optical implant should have a miniaturized size towards the millimeter (mm) scale^34,35^ in order to avoid invasive surgery, infection, inflammation and associated post-surgery trauma. It should also enable highly efficient power transfer and long communication distance to target deep region neurons, especially if aimed towards human applications. One of the most important challenges to implement such implants lies in the energy required to optically activate optogenetic opsins, which is typically up to a few mWs^36^ and much greater than the requirements for conventional electrical stimulation, neurophysiology recording, or data communication. To solve this unmet need, we propose a fully implantable, miniaturized, wireless optical stimulator, using an mm-sized Rx coil optimized at a low resonant frequency of <100 MHz to deliver sufficient power for µLED operation without surpassing the operational temperature increase limit. The light source consists of a single µLED being coupled to a microscale, highly reflective lens to enhance optical throughput and penetration depth of LED illumination for effective epidural neuro-stimulation with minimal invasiveness.

**Fig. 1:**
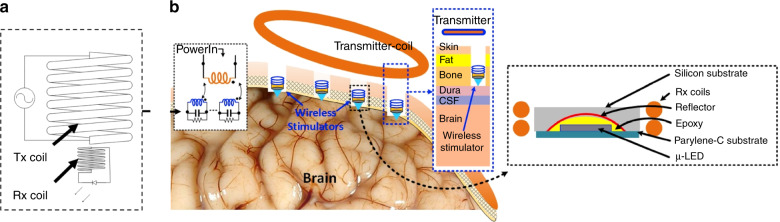
Conceptual diagram of the inductively coupled neuro stimulator system. **a** An overview of the inductive link system. **b** Conceptual diagram of the wirelessly-powered opto neuro-stimulator and its placement over the cortex of the animal brain

Illustrated in Fig. 1 is a conceptual diagram of the proposed single-channel opto-neurostimulator and its potential application on an experimental animal brain. Our proposed system consists of a solenoid transmitter (Tx) coil and a receiver (Rx) unit. The receiver unit consists of a single µLED on a polymer substrate, which is coupled with a silicon micro-reflector (µ-reflector). A solenoid coil is wound around the silicon reflector base and integrated with the µLED as a receiver coil. While being powered by a function generator, the Tx is designed to transmit energy wirelessly to the Rx coil, allowing effective activation of the single channel stimulator. A proof-of-concept prototype for the proposed system is designed and implemented in this work, in which a blue μLED (CREE TR2227tm) with a central wavelength of 465 nm and a surface area of 270 μm × 220 μm is used to optically excite neurons expressing channelrhodopsin. The LED reflector is fabricated from silicon (Si) with dimensions of 1 mm × 1 mm × 500 µm. The integrated reflector-coupled μLED stimulator has overall dimensions of 3 mm × 2 mm. A 2-mm-inner diameter and 9-turn Rx coil is made of copper wires (AWG 34) and wound around the Si base of the μLED source (see the fabricated device in Supplementary Fig. S1). The Tx coil has 6 turns and a 5 mm inner diameter, also constructed in a solenoid configuration (AWG20) using copper wires. To validate the device functionality in vivo, the Tx coil is placed outside of the brain and inductively coupled to the Rx coil, while the Rx coil integrated μLED neuro-stimulator is dropped within a craniotomy cavity on top of the dura mater for epidural optical neuromodulation using the free-floating method^37^.

## Results and discussion

### Fabricated device

**Fig. 2:**
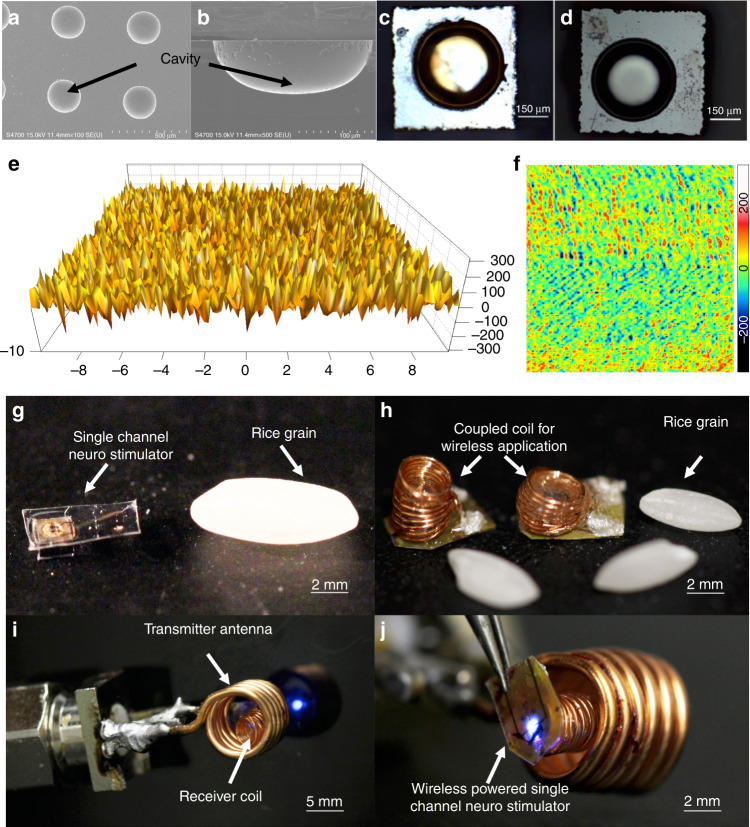
Characterization results and images of an operating prototype. **a**, **b** SEM images of the etched Si cavity. **c**, **d** Post and prior Al coating of the cavity, respectively. **e**, **f** AFM images for quantitative analysis of cavity surface roughness (*x* axis units in μm, *y* axis in nm). **g** Fabricated single-channel opto neurostimulator. **h** Rx-coil coupled single-channel opto neurostimulators. **i**, **j** An opto stimulator powered wirelessly by a Tx coil

Coupling the LED to the micro-fabricated cavity reflector provides a significant boost in optical intensity, as seen in previous studies^38^. Figure 2a, b shows the scanning electron microscopy (SEM) images of a cavity array after isotropic Si etching and cross-section of a single cavity, respectively, where a smooth surface morphology was observed. Figure 2c, d shows silicon cavities after and before a reflective aluminum coating, respectively. Atomic force microscopy (AFM) analysis in Fig. 2e, f shows a small mean roughness (~72 nm) for the etched cavity, confirming the smooth morphology of the cavity resulted from the wet isotropic etching. This roughness, compared with the radius of the cavity (~100 µm), is supposed to create a negligible light scattering, thereby allowing the reflector to optimally enhance the intensity. Figure 2g–j shows a fabricated neurostimulator, and its activation through inductive powering on a benchtop setup.

In spite of the superior biocompatibility of Parylene-C, it does suffer from the potential issue of cracking. As indicated in the previous results^39^ reported by Hsu et al., cracking is more probable when there is a high-temperature process involved such as soldering interconnections (at ~350 °C), or if there is a sudden temperature change associated such as a rapid cooling from a much higher temperature. From our benchtop and in-vivo experiments, we speculate the absence of cracks in the encapsulation due to our utilization of a low melting solder to bond LEDs and coils with the metal pads. The solder liquefies at ~65 °C and imposes very minimal threat of cracking to the Parylene-C layers used. Moreover, as the soldering process is performed in a warm acidic solution (HCl + H_2_O, pH = 1) to maintain the solder liquefaction, it does not involve any rapid cooling from a very high temperature. The stimulator did not undergo any other high thermal process. Further proof of any presence of cracks could be verified by soak testing, which was kept out of scope for this work.

### Finite element simulation

**Fig. 3:**
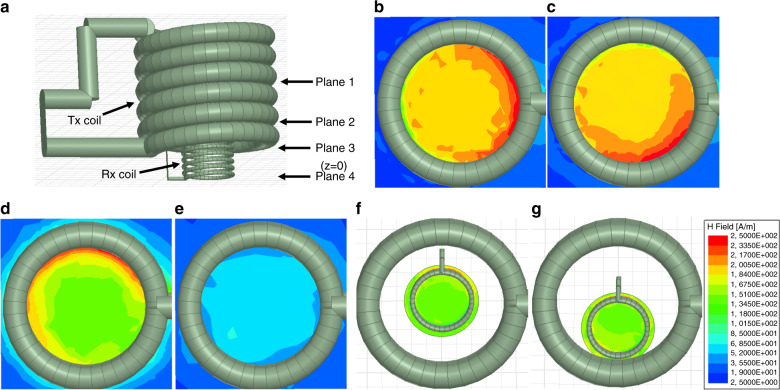
Simulation model for the inductive coupling at cross-section planes. Plane 3 refers to the bottom plane of the Tx coil (z axis displacement, *z* = 0), plane 1, 2, 4 refers to *z* = 2.5 mm, *z* = 1 mm, and *z* = −1 mm, respectively. **a** The simulation model in HFSS. Magnetic flux distributions for **b** plane 1, **c** plane 2, **d** plane 3, and **e** plane 4. Induced magnetic flux by the Rx coil at plane 2, while the Rx is positioned at **f** Tx center and **g** Tx periphery. Units of flux distributions provided in the color map legend

Electromagnetic properties of the inductive link between the Rx and Tx coils were also simulated using the finite element method (FEM) in High Frequency Structure Simulator software (HFSS-Ansys electromagnetics suite 17.2, Ansys). Figure 3a illustrates the HFSS device model of the two-coil telemetry link, and Table 1 provides a set of parameters used to design the Rx and Tx models for FEM simulation. In this study, the dimensions of the simulated Tx coil were similar to those of the fabrication coil. The inner and outer diameters of the Rx coils remained the same as the fabricated devices, while the number of turns was varied to study its effect on the resonant frequency and power transfer efficiency (PTE) of the inductive power link. The pitch between adjacent turns was estimated using the overall length of the solenoid coil and the diameter of the copper wire.

**Table 1:** Simulation parameters

	Wire radius (mm)	Coil radius (mm)	Pitch (mm)	# of turns
Tx	0.406	2.5	1	6
Rx	0.08	1.0	0.275	3, 6, 9, 12, 15

Magnetic flux distribution of the Tx coil was simulated as cross-sectional planes at the mid-point, bottom, and 1 mm below the bottom plane of the Tx. As shown in Fig. 3b, c, a stronger magnetic flux is generated at the periphery of the Tx coil due to the asymmetrical winding of the Tx loops. Consequently, the inductively induced magnetic field in the Rx coil is stronger when the Rx coil is aligned to the periphery of the Tx coil, as shown in Fig. 3f, g. In addition, the magnetic flux is concentrated in the middle plane of the coil and the strength of the flux reduces significantly as the simulated plane approached towards the bottom of the coil, as indicated in Fig. 3b–e.

### Optical properties

**Fig. 4:**
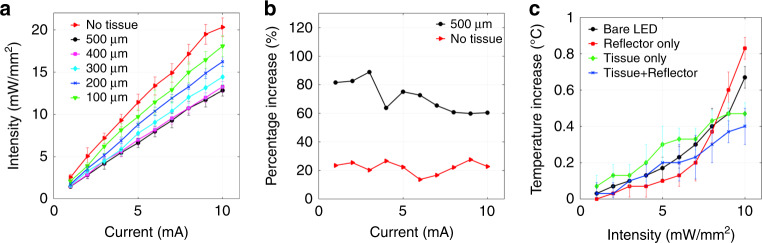
Optical and thermal characteristics. **a** Light penetration through tissue sections with a coupled reflector (*n* = 5), **b** intensity improvement of reflector coupled stimulator compared with a bare μ-LED, and **c** change in temperature for devices stimulating through a 500 μm cortical tissue slice (*n* = 3)

The incorporation of the reflector enhances the intensity while also focusing the light beam to some extent, when compared to a bare µ-LED or a planar mirror, as reported in our previous work^38^. Although an intensity enhancement effectively results in a reduction of illumination range, the results illustrated in Fig. 4 reports the presence of sufficient beam intensity for optogenetic stimulation at a depth of ~500 µm. This phenomenon provides our stimulator with the advantage of reaching deep brain cells in a less invasive way compared to the waveguide or penetrating probe approaches. The focusing effect, which results in the reduction of the lateral illumination range, also enables a potential increase in spatial resolution of optical stimulation, a much-needed capability for neuro-stimulator arrays.

The optical analysis illustrated in Fig. 4 indicates the superior optical performance of the reflector-coupled stimulator. Figure 4a shows the captured intensity after the light passed through a tissue section. Our data indicates that although the intensity reduces heavily with introductions of thicker tissue sections, the threshold (1 mW/mm^2^) for opsin activation^40^ is achieved even for a 500 µm thickness section, establishing an elementary yet efficient method for brain stimulation without tissue invasion. Figure 4b shows the intensity increase in percentage, where a reflector-coupled stimulator was compared with a bare stimulator. Light intensity, along with illumination depth significantly increases by over 60%, which was measured through 500-µm-thick tissue slices. This advantage makes our reflector-coupled stimulator a considerable competitor for deep brain stimulation, capable of optical stimulation from the cortical surface rather than brain tissue invasion^41^.

### Thermal properties

The temperature increase of the reflector-coupled stimulator under different operating conditions is plotted in Fig. 4c, and compared to that of a bare µLED stimulator (i.e., without the integrated µ-reflector component). The addition of a reflector reduces the heat dissipation to the silicon substrate due to thermally insulating materials (i.e., polymer, epoxy, and air) in the reflective cavity, thereby resulting in higher temperature increases. Most importantly, the overall temperature rise of the reflector-coupled stimulators falls below 0.5 °C, complying with the American Association of Medical Instrumentations (ANSI/AAMI) standard limit of 2 °C increase for chronic biomedical implants for neuro-stimulators (ISO-14708-1).

### Electromagnetic properties

**Fig. 5:**
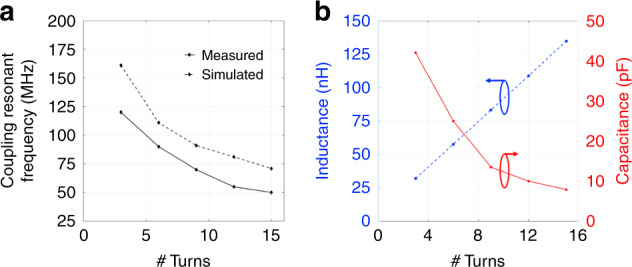
Electro-magnetic characteristics. **a** Resonant frequency of the two-coil inductive link with respect to the number of turns of the Rx coil. **b** Inductance and parasitic capacitance of the Rx coil as functions of the number of turns, calculated using impedance fitting based on the analytical models (see “equivalent circuit design” section)

Figure 5a shows the resonant frequency with respect to the number of turns of the Rx coil. With an increase in the loops, the resonant frequency was found to be decreasing and for ≥9 turns, both our experiments and simulations provide a resonant frequency of <100 MHz. A low resonant frequency is highly desirable to maintain nerve vitality, which effectively minimizes the electromagnetic exposure to living tissues and reduce the risk of radiation^23^. The decrease in resonant frequency is due to the increase in flux linkage that increases with the turns for the Rx. However, increasing the turns or the coil length simultaneously increases the device dimensions and thereby makes it challenging towards fabricating a fully implantable stimulator. The proposed single channel neuro-stimulator allows pulse (~5 ns) stimulation in acute animal studies, which is controlled by the supply frequency of the power source. For devices used in behaving animal studies, miniaturized ASIC chips with built-in pulse modulation function could be integrated to enable optical stimulation in a pulse mode. Our developed fabrication processes are compatible with the conventional semiconductor microfabrication techniques, allowing the integration of µLEDs and reflective cavities with foundry-fabricated CMOS chips. It should be noted that, for experimental purposes, the coils were manually constructed using a winding rod so the actual devices have a slight deviation in pitch and length than the simulation model, resulting in the difference in their resonant frequencies between the simulation and experimental conditions. Figure 5b shows the inductance calculated based on Eq. (1) and the capacitance derived from impedance fitting based on Eq. (5) (see “methods and materials” section).

**Fig. 6:**
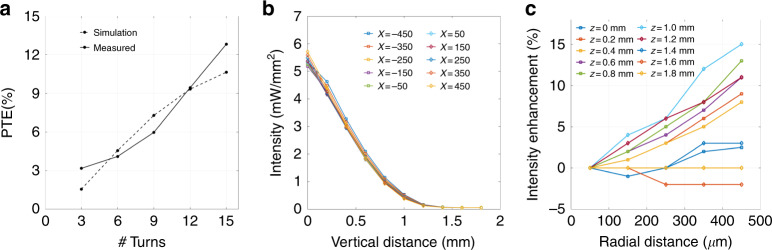
Power transfer efficiency (PTE) and prototype alignment. **a** PTE change with respect to the Rx coil turns. **b** Optical intensity obtained from a 9-loop coiled stimulator under different vertical displacements of the coils. Symbol “x” in (µm) indicates the horizontal position of the Rx center compared with the Tx center, while vertical distance (mm) indicates the displacement from the bottom horizontal plane Rx, compared with the bottom plane of Tx. **c** Intensity enhancement of a 9-loop coiled stimulator under different horizontal displacements of the coils Symbol “z” in (mm) indicates the vertical position of the Rx with respect to the Tx bottom plane, while radial distance (µm) indicates the displacement from the axis of Tx

Figure 6a shows the measured and simulated PTE values with respect to the number of turns of the Rx coil. The measured PTE harmonizes with the simulated model, with a maximum efficiency of 12.82% for a 15-loop implant coil. It is also noted that, given the same Tx coil, the Rx coils with more turns have relatively higher PTE but larger dimensions. Considering the tradeoff between PTE and miniaturization of the implant, we utilized a 9-loop Rx coil in the first generation prototype for the following animal studies. Prior to animal studies, it is critical to understand the effects of the separation and misalignment of the coil link on the electromagnetic coupling performance and the optical properties of the optical stimulator. Therefore, we studied the optical intensity of the stimulation under various vertical and horizontal displacements between the Tx and Rx coils. As shown in Fig. 6b, the optical intensity is higher when the Rx coil of the stimulator is closer to the center region of the Tx coil, while the opsin activation threshold (>1 mW/mm^2^) is achieved at the separation of less than 0.8 mm from the bottom plane of the Tx coil. This phenomenon is supported by our simulation results as plotted in Fig. 3b–d, which demonstrates the gradual diminution of the magnetic flux from the Tx coil center/middle cross-sectional plane towards the bottom plane.

In addition, our results suggest better coupling and hence better optical intensity can be achieved when the Rx coil is intersecting with stronger magnetic fluxes generated by the Tx coil, preferably towards the periphery of the Tx coil. As illustrated in Fig. 6c, a stimulator with a 9-loop coil could achieve as high as 15% intensity enhancement if it is shifted to the periphery from the center of the Tx coil. Our experimental results could be qualitatively supported by the simulations from Fig. 3b–d, where a stronger magnetic flux is observed towards the perimeter of the Tx coil. This concentrated distribution of magnetic flux at the edge, however, becomes evenly allocated across the diameter, when displaced towards the bottom plane. Rx coils with diameter slightly less than the Tx coil would result in higher PTE, however would not be suitable to be implanted on small rodents (such as rats) due to the unavailability of sufficient exposed brain tissue area. One objective of this work was to reduce the form factor of the device to allow multiple implants on an experimental subject. Although a large RX coil was made using copper wire to demonstrate the technology, high quality factor MEMS inductors could be used in the future design to further miniaturize the device^42,43^.

Although the wireless power transfer was operative at a distance of ~1.8 mm from the Tx coil bottom plane, the optical intensity at that distance was insufficient to activate the microbial opsin (ChR2) used in our experiments. However, use of a highly sensitive opsin such as chimera EF (ChEF), that has a peak activation at 0.70 mW/mm^2^ and a steady state activation at 0.46 mW/mm^2^ for ~470–490 nm light^44^ could effectively increase the operating distance of wireless power transfer, compared to the distance achieved in this work by using the normal ChR2.

Furthermore, the working distance could be improved by implementing a three-coil inductive link, where a resonator coil, being larger than the Rx coil in diameter, forms an intermediate link between the Tx and Rx coils to maximize PTE over a long distance in a non-concentric position. The resonator coil would be implanted between the skull and the skin, without wire connection to either the Tx or Rx coil. More details about the 3-coil configuration are described in Kiani et al.^45^ and Mirbozorgi et al.^46^.

### In vivo results

**Fig. 7:**
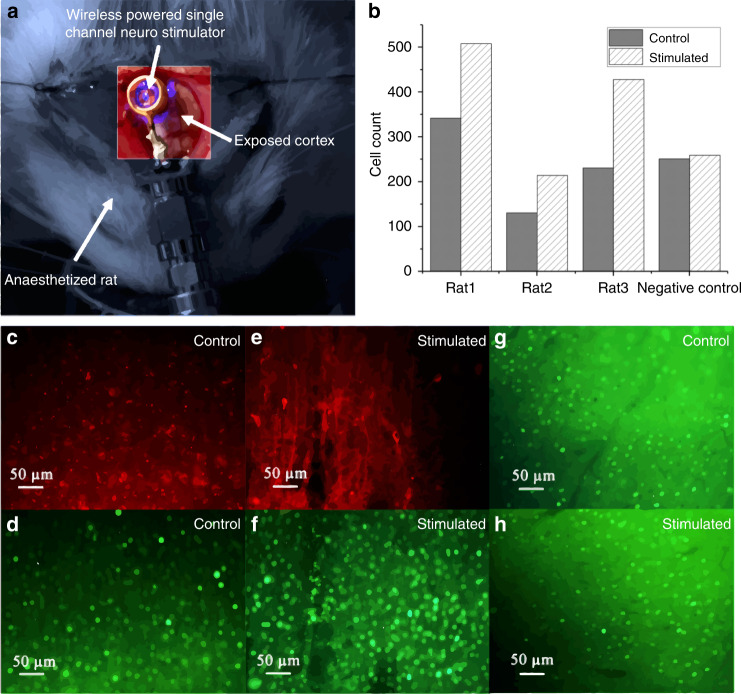
System validation using Immuno-histology. **a** In vivo stimulation using a wirelessly-powered neurostimulator on the V1 of an anaesthetized rat. **b** quantitative representation of c-Fos expressed cells using cell sorting. Fluorecent images of mCherry (**c**, **e**) as well as c-Fos (**d**, **f**) expressions of the control and stimulated cortices, respectively, obtained from the same cortical areas of the same transfected animal. **g**, **h** c-Fos expressions of the control and stimulated cortices, respectively, obtained from a non-transfected animal

In vivo experiments were performed on three (*n* = 3) transfected rats following the protocols mentioned in “in vivo experiment” section. Figure 7a shows a 9-loop coiled stimulator placed over one V1 lobe and coupled to a Tx coil. The other V1 lobe of the same animal was used as a control. Immunochemical analysis was performed on the post perfusion fixed brain tissue to identify the presence of mcherry and c-Fos biomarkers, in order to validate the efficacy of the cell transfection and optical stimulation, respectively. Existence of mcherry expressed cells (in red) are visible, as seen in Fig. 7c. Figure 7e demonstrates the effectiveness of our viral transfection process, for both the control and stimulated lobes on an experimental animal. Figure 7d, f, on the other hand, provides the evidence of c-Fos (in green) expressed cells in both the control and stimulated lobes of the transfected animal. The stimulated V1 lobe (Fig. 7f) has more cells expressing c-Fos as compared to the control side (Fig. 7d), suggesting stronger neural activity induced by optical stimulation. It should be noted that due to the natural activity of the visual cortex, there will be a population of cells expressing c-Fos even at the absence of any stimulation, while a larger population of cells are expected to show c-Fos expression as a result of the optogenetic stimulation. To provide a robust evidence towards the efficacy of the optogenetic stimulation, we repeated the above optical stimulation on a naive animal without virus transfection, and the cortical tissues were processed immuno-biologically using the above method to analyze the c-Fos expression of the stimulated lobe versus the non-stimulated lobe. As shown in Fig. 7g, h, there is no visible significant difference in the c-Fos expression between the stimulated lobe and the non-stimulated control side. The presence of c-Fos expression in both the simulated and non-stimulated lobes is attributed to the spontaneous neural activity in the animal cortex. This result demonstrates that the elevated c-Fos expression in the virus transfected cortex is indeed induced by LED stimulation, eliminating the possibility of other interferences, such as heat or tissue injuries.

A quantitative measurement of the expressions, such as cell sorting, was performed based on the fluorescent images of the tissue sections. Using an in-house Matlab script, computer vision techniques allowing basic morphological operations along with watershed algorithm was utilized to define cell segmentation and an approximate count of cells expressing c-Fos. In concert with the qualitative data, Fig. 7b indicates that the transfected cortices with optical stimulation show a significant increase in the cells expressing c-Fos, and this is consistent among the three subjects. There is a negligible difference in cell activity between control and stimulated cortices of a non-transfected naive animal, supporting our claim of increased cell activity due to the applied stimulation.

Supplementary Table S1 lists the specifications of some recent advances in the development of inductive link battery-free wireless optical neurostimulators and our work. The comparison shows a competitive advantage of our stimulator as a fully implantable biomedical neurostimulators with very lightweight, compact size (mm scale), and low operating frequency.

## Methods and materials

### Equivalent circuit design

**Fig. 8:**
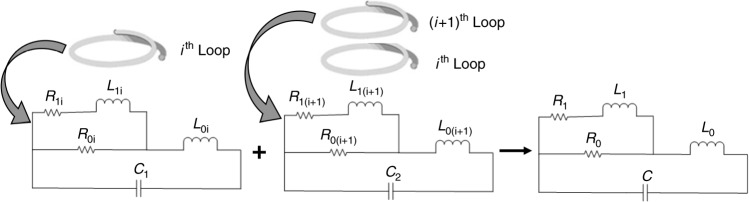
Analytical model for the inductive coupling link. Equivalent circuit model for the Rx coil

Distributed resistive effects from the finite resistivity of the conductor, along with inductive effects from the coiled copper conductor, and dielectric effects from the insulation around the conductor altogether constitute the electrical properties of the Rx coil. An approximation of the electrical characteristics due to the effects of these components is of great importance, as they determine the efficiency and effectiveness of the inductive link towards a successful operation of the whole setup. To facilitate the design of the microcoil, we studied an equivalent circuit model of the Rx coil that provides an estimation of the distributed passive components. As shown in Fig. 8, *R*_0*i*_ and *L*_0*i*_ are the DC resistance and self-inductance of the *i*-loop wire within a multi-loop coil, respectively. *R*_l*i*_ and *L*_l*i*_ are estimated to be in effect by the tendency of alternating current being distributed within the conductor, commonly known as the skin effect. The distributed circuit model in Fig. 8 can be simplified using a combined lump model, where *R*_0_, *R*_1_, *L*_0_, and *L*_1_ represents the lumped resistances and inductances responsible for the impedance change with frequency while considering the skin effect^47^, and the capacitor C is the total effective capacitance across the coil. The self-inductance *L*_0_ and the DC resistance *R*_0_ could be calculated from the following equations:

1 $$L_0 = \frac{{N^2\mu A_c}}{{l_c}}$$

2 $$R_0 = \frac{{\rho D}}{T}$$

where, *N* is the number of turns of the coil, *µ* is the permeability of the core, *A*_*c*_ is the cross-sectional area of the coil, and *l*_*c*_ is the length of the solenoid coil. While calculating the DC resistance, *ρ* is the resistivity of the conductor metal (in this work, copper), *D* being the total length of the coiled conductor wire, and *T* is the cross-sectional area of the used conductor wire. The skin effect components, *R*_1_ and *L*_1_ could be represented by the following equations:

3 $$L_1 = \frac{{L_0}}{{\alpha _L}}$$

4 $$R_1 = \alpha _RR_0$$

where $\alpha _L = 0.315\alpha _R$ and $\alpha _R = \frac{{0.53 \ast {\text{wire}}\;{\text{radius}}}}{{\delta _{\text{max}}}}$. The skin depth in conductors, *δ*_max_, is represented by $\delta _{\text{max}} = \sqrt {\frac{2}{{\omega \mu \sigma }}}$, where *ω* is the resonant frequency, *μ* is the magnetic permeability in vacuum, and *σ* is the conductivity of the conductor metal. The constant term 0.53 is derived from the following equation:

$$C = \frac{r}{{2r - \delta _{\text{max}}}}$$

The impedance of the lumped equivalent circuit could be represented by the following equation:

5 $$Z = \frac{{R_1R_0 - \omega ^2L_1L_0 + j\omega (R_1L_0 + R_0L_1 + R_0L_0)}}{{R_1 + R_0 + j\omega \left( {L_1 + R_1R_0C - \omega ^2L_1L_0C} \right) - \omega ^2C(R_1L_0 + R_0L_1 + R_0L_0)}}$$

Given the theoretical inductances and resistances that are derived from Eqs. (1)–(4), the parasitic capacitance *C* of the coil can be estimated based on the measured impedance by curve fitting, as shown in Fig. 5b.

### Device fabrication, assembly, and packaging

The fabrication of the stimulator started by depositing 10 µm Parylene-C on a 3-inch Si wafer. Parylene-C was used due to its ability to act as a biocompatible thin film substrate that has a superior optical transparency. The transparency allows the maximum collection of the reflected light beams compared to an opaque substrate. A 500 nm copper (Cu) film on a 5 nm titanium (Ti) adhesion layer was deposited on the Parylene-C substrate using a thermal evaporator (Edward Auto306, Edwards). Copper is selected as a metallization layer in the proof-of-concept prototypes because of its combination of desired properties, including high electrical and thermal conductivity as well as low cost. With the Parylene encapsulation, the Cu metallization layer is unlikely to be oxidized or corroded. For further improvement and optimization of the device, noble metal, such as thin film gold (Au), could be deposited on the Cu layer to minimize the possibility of oxidation, corrosion, and toxicity effects. A photoresist mask (Shipley S1813, MICROCHEM Corp.) was patterned via ultraviolet (UV) lithography. After that, Cu and Ti thin films were deposited and patterned chemically using Cu etchant (Ferric Chloride by MG chemicals) and hydrofluoric acid (HF), respectively, to form the metal pads and traces for µLED and coil assembly. After removing the photoresist mask in acetone and rinsing the wafer in isopropyl alcohol (IPA) and deionized (DI) water, the metal patterns were encapsulated with 5 µm Parylene-C, followed by oxygen plasma etching of Parylene-C with 58 sccm O_2_ at 250 W (PX-250, Nordson March) to create the contact vias on the µLED and coil bonding pads, using a photoresist mask. Commercially available blue μLEDs (TR2227tm, CREE Inc.) were manually aligned on the exposed metal contacts and bonded with low melting point (LMP) solder (melting point at ~62 °C, 144 ALLOY Field’s Metal, Rotometals, Inc.).

The µ-reflector was fabricated in a separate step, initiated with thermal evaporation of 500 nm Cu on a 300-nm thick, commercial low-pressure chemical vapor deposited (LPCVD) nitride-on-silicon wafer (University wafers, Boston). Because of its low etching rate under SF6 plasma^48^, Cu was used as a mask to pattern the LPCVD nitride with 20 sccm SF6 at 250 W RF power for 45 s. After nitride patterning, the Cu mask was removed using Cu etchant, followed by DI water rinse. The nitride masked Si wafer was then submerged in an HF and nitric acid (HNO_3_) (1:9 by volume) solution for 60 min, which isotropically etches Si to form hemispherically shaped microcavities^49,50^. After that, the nitride mask was removed using hot phosphoric acid (H_3_PO_4_) at 75 °C and the wafer was rinsed with DI water. Finally, a 100 nm aluminum (Al) layer was deposited in the cavities using thermal evaporation to form a highly reflective surface for collecting the µLED backside emission and focusing the diverged light beams.

For device assembly, the wafer with the reflector array first was diced into 1 mm × 1 mm chips containing a cavity on each. Manual alignment and positioning were performed to couple the μLED substrate and the Al-coated cavity reflector under an optical microscope while bonding using a medical grade epoxy (Atom adhesives AABond-FDA2), and cured at room temperature (25 °C) for 24 h. Fabricated separately, the micro reflector dice was initially integrated with the Parylene-C substrate, followed by the integration of the mm-scale coil Rx coil. The area of the coil being slightly larger, fitted around the dice for component integration completion. Finally, the assembled device was completely encapsulated using 12 µm Parylene-C. Supplementary Fig. S1 provides a simplified process flow for the fabrication and integration of the implantable neurostimulator.

### Device characterization

#### Optical property measurement

The surface roughness of the Al-coated reflective layer was measured using a Surface Profilometer (NanoMap-500LS) as shown in Fig. 2. The average surface roughness of the metal coated cavity was ~15 nm, indicating minimal scattering of the reflected light. The light intensity of the as-fabricated neuro stimulator penetrating through different thicknesses brain tissue was measured using a Newport 818-SL optical detector coupled with an OD3 attenuator and Newport 843-R series optical power meter, and data was compared with that of a bare µLED.

The brain tissue slices with thicknesses of 100–500 µm were prepared from fixed cortical tissues of rats. The animals were euthanized with an Intra-peritoneal injection of heparin followed by an overdose of pentobarbital sodium. Then, they were perfused transcardially with 4% paraformaldehyde in 0.1 M phosphate buffer (pH 7.4). Post perfusion, the brain was exposed and the head immersed in the same fixative at 4 °C for at least 24 h then in phosphate-buffered saline (PBS). Finally, the brain tissues were cut into serial 100–500 µm coronal sections using a Vibratome (Lancer).

#### Thermal property measurement

The temperature profile of the stimulator during continuous operation provides critical information to study the effect of trapped heat due to the silicon reflector, and also gives an insight on how tissue sections might be able to help in dissipation of the heat from the stimulator to surrounding medium. Hence, we measured the thermal energy distribution of the device while stimulating over a 500 µm rat cortical tissue slice using a high resolution, thermal imaging camera (FLIR E6, FLIR® Systems, Inc.). The temperature increase was quantified with respect to the ambient temperature (22 °C) and compared with that of a bare µLED.

#### Electromagnetic properties measurement

The electromagnetic properties of the Tx coil, Rx coil, and 2-coil inductive link were characterized using a microwave network analyzer (Keysight N5227A PNA, Keysight Technologies). The two-port S parameter (S21) for the 2-coil coupling link was measured to calculate the change in PTE with respect to the number of turns of the Rx coil. The resonance frequency of the coupling was also measured from the S parameter. The experimental results were compared with the simulated results from “finite element simulation” section. Furthermore, the impedance for the Rx coil was measured using an impedance analyzer (HP 4192, Hewlett Packard), and the obtained data was used to calculate the parasitic capacitance of the Rx coil based on Eq. (5). For all the measurements, the Tx coil had an inner diameter of 5 mm, outer diameter of 5.08 mm, and 6 turns.

#### *In vivo* experiment

##### Animal handling and viral transfection

Adult female Sprague Dawley rat (300–400 g) were used in all experiments. Rats were housed in the animal facility of the Michigan State University, USA under standard conditions at a constant temperature of 22 °C supplied with food and water. Virus injection and device implantation were performed using sterile surgical protocols approved by the Institutional Animal Care and Use Committee (IACUC) at Michigan State University. All efforts were made to minimize the number of animals used and prevent or ameliorate their suffering.

For virus injection, rats were fixed on a stereotaxic frame while anesthetized with 2–4% isoflurane and oxygen mixture. A 3~4 cm incision was made in the skin overlying the skull and small cavities were made bi-laterally on the skull to get access to the primary visual cortex (V1, lateral: 3.6 mm, anteroposterior: 6.3 mm relative to Bregma) using a precision surgical drill. Channelrhodopsin-2 expression was induced by administration of a viral solution of AAV-hSyn-hChR2 (H134R)-mcherry (10^12^–10^13^ genome/mL). The process followed a 1 µL aliquot being delivered to each cavity by slow pressure injection from a Hamilton syringe. One (1) μL aliquot was delivered through two loads, with 0.5 µL per load followed by a 10 min wait after each load to allow sufficient diffusion of the virus to the surrounding tissues. After the syringe was retracted, the cortical cavity was filled with surgical wax and the craniotomy wound was sutured. Prior to suturing the skin, the cortex opening was covered with Gel foam. The animals were given appropriate postoperative care (analgesic, fluids) and placed on the heat pad. The animals were returned to the facility and housed separately after recovery from anesthesia, and prevention of infection and relief from surgical discomfort was ensured by providing topical antibiotics and subcutaneous pain medication, respectively.

For device implantation, 2 weeks’ post-injection, the transfected rat was placed back onto the stereotaxic apparatus and underwent the anesthetic process mentioned previously. A unilateral craniotomy was performed by opening a small hole on top of both the right and left V1 lobes while the dura remained intact. A wireless neuro-stimulator was placed in the skull opening. For inductive power transmission, the Tx coil was aligned over the implant and driven by a function generator (AFG3102, Tektronix) with a sinusoidal voltage of 10 Vp-p and a resonant frequency <100 MHz. During the optical stimulation, one V1 lobe was stimulated continuously for 55 min, while the other was considered as a control. It is to be noted that during the craniotomy, selective removal of the skull allowed sufficient lateral opening to place the stimulator on the dura as seen from Fig. 7a, and the dimensions of the Rx coil were as selected to optimize the available area (post craniotomy) and the PTE. The animal skin having elastic properties, would require suturing back to implement a complete implantation of the as-fabricated stimulator.

After the stimulation, the rat was given a 75–90 min survival period. Upon completion of the experiments, animals were euthanized with pentobarbital sodium and then perfused transcardially with 4% paraformaldehyde fixative for histological studies. The brain tissue was post-fixed overnight at 4 °C in the same solution. Tissue sections (500 μm thickness) were cut in chilled 0.1 M phosphate buffer solution and were stored in 24-well tissue culture plates for post-immuno-histology chemical processing. The processed sections were later mounted on microscope glass slides while covering by coverslips with an anti-fade solution for c-Fos (an activity-dependent biomarker) expression. Microscope images were taken using a fluorescent microscope (Nikon MICROPHOT-FXA) to analyze the c-Fos and m-Cherry expressions.

## Conclusion

In this paper, we have designed, fabricated, and characterized a reflector-coupled, wirelessly-powered single-channel optical neuro-stimulator with an mm-sized receiver coil for untethered optogenetic neuromodulation. The optical analysis shows that our reflector-coupled stimulator enables over 60% performance improvement when compared to a bare μ-LED stimulator, simultaneously surpassing the required effective optogenetic activation intensity threshold. The temperature increase of the stimulator is less than 2 °C, well below the safety limit for biomedical implants. The performances of the two-coil telemetry link were studied using analytical circuit models, FEM simulation, and experimental approaches. Inductive coupling between the Tx and a prototype Rx coil was optimized at a <100 MHz carrier frequency, providing a convincing PTE while maintaining small geometries of the coils. The efficacy of optical neuro-stimulation was demonstrated by in vivo experiments on the visual cortex of an anesthetized rat, using qualitative and quantitative immunohistochemical analysis. The increased expression of activity-dependent biomarkers upon optical stimulation establishes a clear evidence of upregulated cell activity due to the optogenetic stimulation.

## Supplementary information

Supplementary material

Supplementary Table
