# Supplementary material for: Inductively coupled, mm-sized, single channel optical neuro-stimulator with intensity enhancer
Source: Microsyst Nanoeng. 2019 Jun 3;5:23. doi: 10.1038/s41378-019-0061-6 (PMC6545326; doi:10.1038/s41378-019-0061-6)
Supplement: Supplementary file 1 — Supplementary material [file 41378_2019_61_MOESM1_ESM.doc]

**Inductively Coupled, mm-Sized, Single Channel Optical Neuro-Stimulator with Intensity Enhancer**

Wasif Khan^1^, Yaoyao Jia^2^, Fatma Madi^3^, Arthur Weber^3^, Maysam Ghovanloo^2^ and Wen Li^1^

^1^Department of Electrical and Computer Engineering, Michigan State University, East Lansing, USA

^2^School of Electrical and Computer Engineering, Georgia Institute of Technology, Atlanta, USA

^3^Department of Physiology, Michigan State University, East Lansing, USA


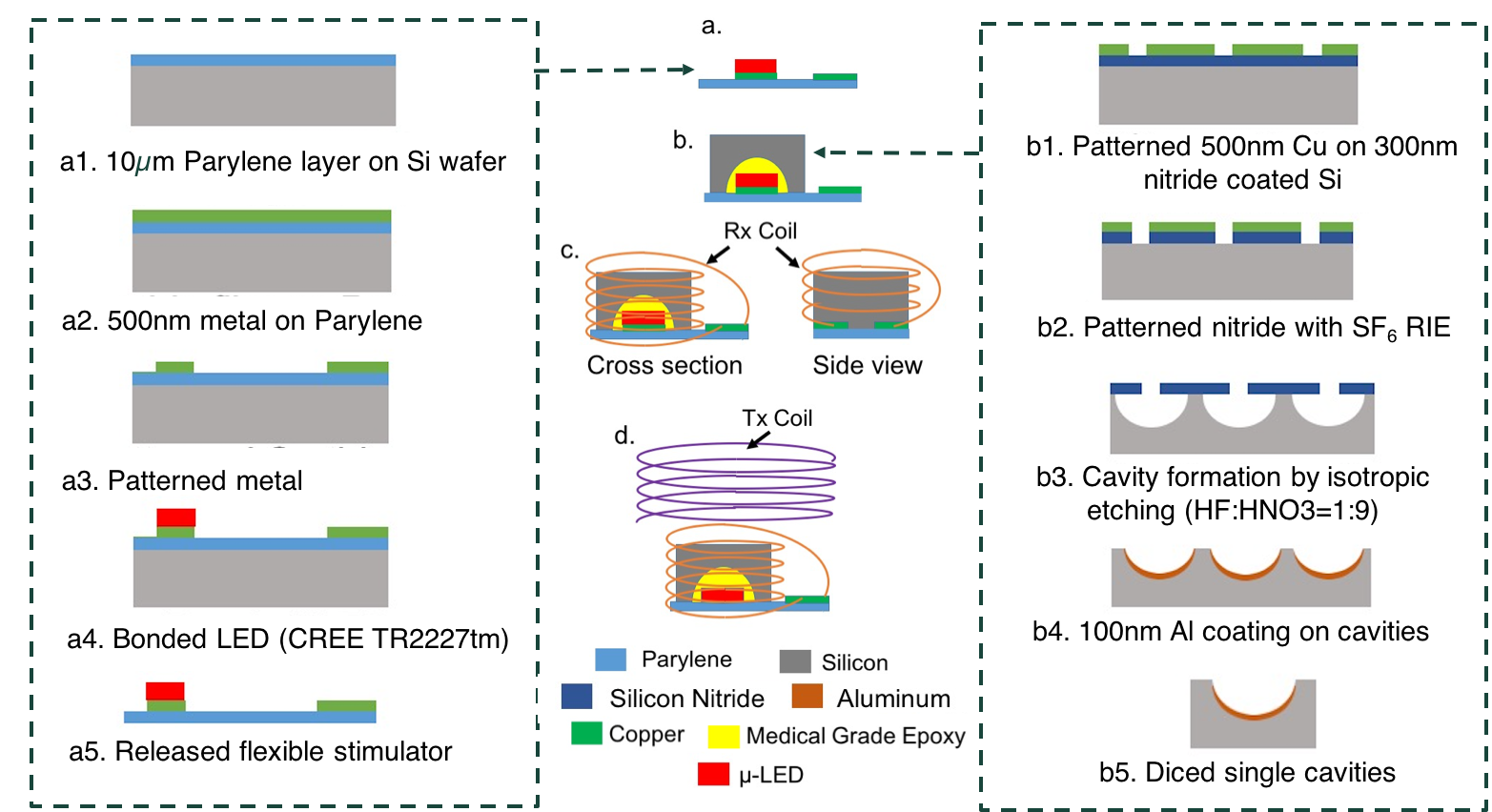


*S1. Simplified fabrication and integration process flow*

Supplementary Table S1. Comparison of our work and neurostimulators reported by other groups

| Work | Architecture | Substrate | Weight  (mg) | Dimensions (mm) | Frequency | channels | Available intensity (mW/mm^2^) |
| --- | --- | --- | --- | --- | --- | --- | --- |
| Kim et al ^1^ | Discrete device | Flexible/rigid PCB | 700 | 14x12.5x3 | 910MHz | 4 | 17.7 |
| Montgomery et al ^22^ | Discrete device | Rigid PCB | 20-50 | 2x2x2 | 1.6 GHz | 1 | 25.8 |
| Shin et. al. ^37^ | Discrete device | Flexible PCB | 30 | 10mm  diameter, thickness<1.3mm | 13.6 MHz | 1,2 | 100 |
| Aldaoud et al ^45^ | Discrete Device | Rigid PCB | 1000 | 5x2.5x2.5 | 20, 13.4/15.6 MHz | 1,2,16 | _ |
| Park et al.^46^ | Discrete device | Rigid PCB | 70 | 2.4x3.5x8.5 | 1.6-2.5 GHz | 4 | _ |
| Park et al ^38^ | Discrete device | Stretchable Polymer | 16 | 3.8x6x0.7 | 2.0-2.5 GHz | 1 | 10 |
| Lee et al ^47^ | LSI | Stacked rigid PCB | 1600 | 12x7x11 | 2.4GHz | 1 | 27 |
| **This work** | **Discrete Device** | **Flexible PCB** | **20** | **2.8x4.2x2.1** | **<100 MHz** | **1** | **5.8** |
