# Supplementary figures and images for: Inductively coupled, mm-sized, single channel optical neuro-stimulator with intensity enhancer
Source: Microsyst Nanoeng. 2019 Jun 3;5:23. doi: 10.1038/s41378-019-0061-6 (PMC6545326; doi:10.1038/s41378-019-0061-6)

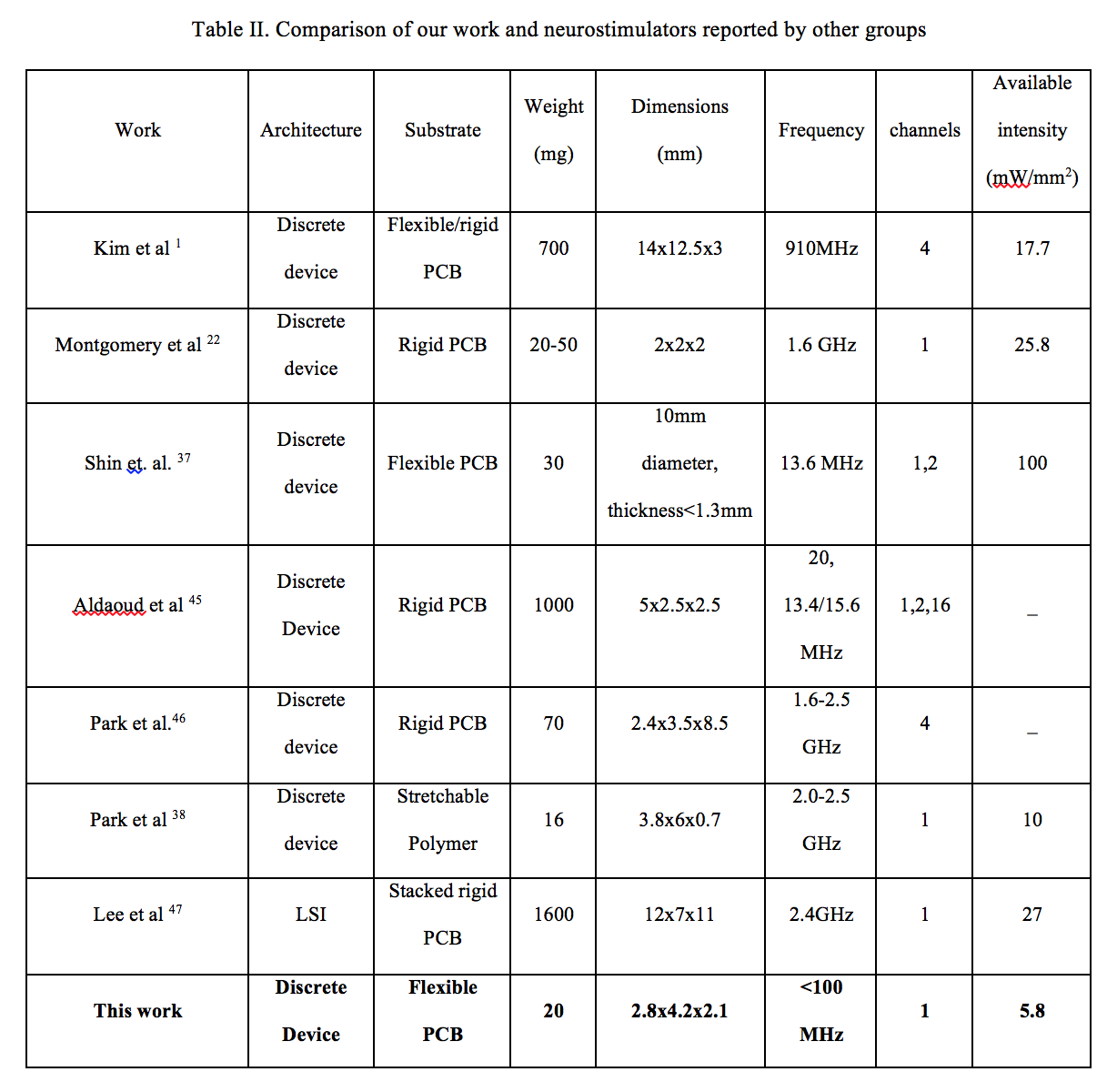

Supplement: Supplementary file 2 — Supplementary Table [file 41378_2019_61_MOESM2_ESM.tif]
